# Ascorbic acid-dependent gene expression in *Streptococcus pneumoniae* and the activator function of the transcriptional regulator UlaR2

**DOI:** 10.3389/fmicb.2015.00072

**Published:** 2015-02-11

**Authors:** Muhammad Afzal, Sulman Shafeeq, Oscar P. Kuipers

**Affiliations:** ^1^Department of Molecular Genetics, Groningen Biomolecular Sciences and Biotechnology Institute, University of GroningenGroningen, Netherlands; ^2^Department of Bioinformatics and Biotechnology, Government College UniversityFaisalabad, Pakistan; ^3^Department of Microbiology, Tumor and Cell Biology, Karolinska InstitutetStockholm, Sweden

**Keywords:** ascorbic acid, UlaR2, pneumococcus, *ula2* operon, AdcR, Zinc

## Abstract

In this study, we have explored the impact of ascorbic acid on the transcriptome of *Streptococcus pneumoniae* D39. The expression of several genes and operons, including the *ula* operon (which has been previously shown to be involved in ascorbic acid utilization), the AdcR regulon (which has been previously shown to be involved in zinc transport and virulence) and a PTS operon (which we denote here as *ula2* operon) were altered in the presence of ascorbic acid. The *ula2* operon consists of five genes, including the transcriptional activator *ulaR2*. Our β-galactosidase assay data and transcriptome comparison of the *ulaR2* mutant with the wild-type demonstrated that the transcriptional activator UlaR2 in the presence of ascorbic acid activates the expression of the *ula2* operon. We further predict a 16-bp regulatory site (5′-ATATTGTGCTCAAATA-3′) for UlaR2 in the P*ula2*. Furthermore, we have explored the effect of ascorbic acid on the expression of the AdcR regulon. Our ICP-MS analysis showed that addition of ascorbic acid to the medium causes zinc starvation in the cell which leads to the activation of the AdcR regulon.

## Introduction

*Streptococcus pneumoniae* is an opportunistic Gram-positive human nasopharyngeal pathogen that causes millions of deaths each year worldwide due to pneumococcal infections like pneumonia, meningitis, bacteremia, otitis media (Mitchell, 2003; Kadioglu et al., 2008; O'Brien et al., 2009). It relies on different carbohydrate sources for its survival in the host, although there is a limited availability of free carbohydrate sources in its initial colonization niche, the nasopharynx (Ispahani et al., 2004; Iyer et al., 2005; Kadioglu et al., 2008; Bidossi et al., 2012; Buckwalter and King, 2012). However, the pneumococcus can make use of the complex glycoconjugates available in the host environment to obtain energy required for colonization (King et al., 2006; Burnaugh et al., 2008; Marion et al., 2011).

Like many other bacteria, glucose is the preferred carbon source for *S. pneumoniae*, but due to the presence of different sugar-specific PTSs (phosphotransferase systems), it can make use of various other carbon sources including ascorbic acid (Cochu et al., 2003; Bidossi et al., 2012). Many bacteria have been shown to ferment ascorbic acid and use it as a carbon source (Yew and Gerlt, 2002; Bidossi et al., 2012; Mehmeti et al., 2013). Ascorbic acid enters the cell through the ascorbate-specific PTS (UlaABC) that putatively carries out the assimilation of ascorbic acid in many bacteria including *Escherichia coli* (Yew and Gerlt, 2002; Campos et al., 2004) and *Klebsiella pneumoniae* (Campos et al., 2008). In our previous study, we showed that *S. pneumoniae* also harbors the *ula* operon, consisting of nine genes, that shares high homology with the *ula* gene cluster responsible for the assimilation of ascorbic acid in *E. coli* and *K*. *pneumoniae* (Afzal et al., 2015). However, there are a number of variations in the organization and regulation of the *ula* locus in *S. pneumoniae* compared to *E. coli* and *K. pneumoniae*. One major difference is the mode of regulation of the *ula* operon. In *S. pneumoniae*, the *ula* operon is activated by transcriptional regulator UlaR in the presence of ascorbic acid, whereas in *E. coli* and *K*. *pneumoniae* the *ula* gene cluster is repressed by UlaR in the absence of ascorbic acid (Campos et al., 2004, 2008; Afzal et al., 2015).

*K*. *pneumoniae* and *E. coli* are reported to have two systems (*ulaABCDEFG* and *yiaKLX1X2PQRS*) for the transport and utilization of ascorbic acid (Campos et al., 2007, 2008). This may also suggest the presence of another ascorbic acid-specific PTS in *S. pneumoniae*. To explore this, we investigated the effect of ascorbic acid on the whole transcriptome of *S. pneumoniae*. We did find another PTS operon (denoted as *ula2* operon) that was upregulated in the presence of ascorbic acid. The *ula2* operon consists of five genes, including one coding for the transcriptional regulator UlaR2 and has high sequence identity with the *ula* operon of *S. pneumoniae* D39. We further demonstrated with the help of microarray analysis and β-galactosidase assays that the transcriptional regulator UlaR2 acts as a transcriptional activator of the *ula2* operon in the presence of ascorbic acid. We have also predicted the putative regulatory site (5′-ATATTGTGCTCAAATA-3′) in the promoter region of the *ula2* operon for the transcriptional activator UlaR2. Furthermore, we have also shown through β-galactosidase assays and ICP-MS (Inductively Coupled Plasma Mass Spectrometry) analysis that ascorbic acid in the medium leads to zinc starvation in the cell, which results in the upregulation of the AdcR regulon in the presence of ascorbic acid.

## Materials and methods

### DNA techniques, bacterial strains, growth conditions, and β-galactosidase assays

All DNA manipulation techniques, growth conditions and media were the same as described previously (Afzal et al., 2014), unless indicated otherwise. β-Galactosidase assays were performed as described before (Israelsen et al., 1995), using cells that were grown in M17 medium with appropriate carbon sources as mentioned in the Results section (Terzaghi and Sandine, 1975). For β-galactosidase assays, derivatives of *S. pneumoniae* D39 were grown in M17 medium supplemented with different sugars (arabinose, ascorbic acid, cellobiose, dextrose, fructose, fucose, galactose, glucose, lactose, maltose, mannose, melibiose, sorbitol, sucrose, trehalose, and xylose) with concentrations mentioned in the Results section and harvested in their respective mid-exponential phase of growth. For selection on antibiotics, the medium was supplemented with the following concentrations of antibiotics: tetracycline, 2.5 μg/ml for *S. pneumoniae*; and ampicillin, 100 μg/ml for *E. coli*. All bacterial strains used in this study were stored in 10% (v/v) glycerol at −80°C. Bacterial strains and plasmids used in this study are listed in Table 1. Primers used in this study are based on the DNA sequence of the D39 genome (Lanie et al., 2007) and listed in Table 2.

**Table 1:** **List of strains and plasmids used in this study**.

**Strain/plasmid**	**Description**	**Source**
***S. PNEUMONIAE***		
D39	Serotype 2 strain, *cps 2*	Laboratory of P. Hermans
Δ*ulaR2*	D39 *ulaR2* null mutant	This study
MA401	D39 Δ*bgaA*::P*ula2*-*lacZ*; Tet^R^	This study
MA402	Δ*ulaR2* Δ*bgaA*::P*ula2*-*lacZ*; Tet^R^	This study
SS201	D39 Δ*bgaA*::P*adcR*-*lacZ*; Tet^R^	Shafeeq et al., 2011a
SS202	D39 Δ*bgaA*:: P*adcAII*-*lacZ*; Tet^R^	Shafeeq et al., 2011a
SS203	D39 Δ*bgaA*:: P*phtA*-*lacZ*; Tet^R^	Shafeeq et al., 2011a
SS204	D39 Δ*bgaA*::P*phtB*-*lacZ*; Tet^R^	Shafeeq et al., 2011a
SS205	D39 Δ*bgaA*::P*phtE*-*lacZ*; Tet^R^	Shafeeq et al., 2011a
***E. COLI***		
EC1000	Km^R^; MC1000 derivative carrying a single copy of the pWV1 *repA* gene in *glgB*	Laboratory collection
**PLASMIDS**		
pPP2	Amp^R^ Tet^R^; promoter-less *lacZ*. For replacement of *bgaA* with promoter *lacZ* fusion. Derivative of pPP1	Halfmann et al., 2007
pORI280	Erm^R^; *ori^+^ repA^−^;* deletion derivative of pWV01; constitutive *lacZ* expression from P32 promoter	Leenhouts et al., 1998
pMA401	pPP2 P*ula2*-*lacZ*	This study
pSS101	pPP2 P*adcR*-*lacZ*	Shafeeq et al., 2011a
pSS102	pPP2 P*adcAII*-*lacZ*	Shafeeq et al., 2011a
pSS103	pPP2 P*phtA*-*lacZ*	Shafeeq et al., 2011a
pSS104	pPP2 P*phtE*-*lacZ*	Shafeeq et al., 2011a
pSS105	pPP2 P*phtB*-*lacZ*	Shafeeq et al., 2011a

**Table 2:** **List of primers used in this study**.

**Name**	**Nucleotide sequence (5′ → 3′)**	**Restriction site**
ulaR2-1	CATGGGATCCCTACGGTTCAATCCCTTGC	BamHI
ulaR2-2	GCATCTCTCTGCTCTTCCG	–
ulaR2-3	CGGAAGAGCAGAGAGATGCGTAGACAGCAAGTTGGCTAGT	–
ulaR2-4	CATGGAATTCTGTATGGGTCGATATGATCACT	EcoRI
ulaR2-F	CATGGAATTCCGTAGAAGGCTTATTGAAGTC	EcoRI
ulaR2-R	CATGGGATCCGTGCATATCCTATCAACACC	BamHI
**RT PCR PRIMERS**		
IR-I-1	ACTATGATGACATTAAAATG	–
IR-I-2	ATACAGATTCAATATTCATC	–
IR-II-1	AGTATTATTGATATGGATGA	–
IR-II-2	AGCTGGTGTACTAACAATAT	–
IR-III-1	TCTAGCAGTTATGTTTGGAG	–
IR-III-2	AATCGGATGTTAGTCGCAAA	–
IR-IV-1	GATTAAGTCAGGAATTGGAGG	–
IR-IV-2	GCTTCTAAGACTACTATATC	–

### Construction of the *ulaR2* mutant

A markerless *ulaR2* mutant (Δ*ulaR2*) was constructed in the *S. pneumoniae* D39 strain using the pORI280 plasmid, as described before (Kloosterman et al., 2006). Primer pairs ulaR2-1/ulaR2-2 and ulaR2-3/ulaR2-4 were used to generate PCR fragments of the left and right flanking regions of *ulaR2*, respectively. Mutants were further examined for the presence of the *ulaR2* deletion by PCR and DNA sequencing.

### Construction of transcriptional *lacZ*-fusions

An ectopic *lacZ*-fusion to the *ulaR2* (*spd_1961*) promoter was constructed in the integrated plasmid pPP2 (Halfmann et al., 2007) with the primer pairs mentioned in Table 2, yielding pMA401. This construct was subsequently introduced into the wild-type and Δ*ulaR2* strains (via double crossover in the *bgaA* locus), resulting in strains MA401 and MA402, respectively. All plasmid constructs were checked by PCR and DNA sequencing.

### Inductively coupled plasma mass spectrometry (ICP-MS) analysis

To determine the intracellular concentration of metal ions by ICP-MS analysis, *S. pneumoniae* D39 was grown in 15 ml of M17 with and without 10 mM ascorbic acid. The cells were harvested at the mid-exponential growth phase. Cultures were centrifuged and washed (at 4°C) twice with M17 medium, and twice with phosphate-buffered saline (PBS). The cell pellets were dried overnight in a vacuum concentrator at room temperature. The ICP-MS analysis was performed on the cells as described before (Jacobsen et al., 2011; Shafeeq et al., 2011a). Results were expressed as μg of metal ions per g dry weight of cells.

### Microarray analysis and reverse transcription (RT)-PCR

For microarray analysis of the effect of ascorbic acid on the transcriptome of *S. pneumoniae*, wild-type D39 was grown in three independent replicates in M17 and in AM17 (10 mM ascorbic acid + M17) medium, and harvested at their respective mid-exponential growth phase.

To analyze the effect of the *ulaR2* deletion on the transcriptome of *S. pneumoniae*, D39 wild type and Δ*ulaR2* strains were grown in three independent replicates in AM17 (10 mM ascorbic acid + M17) medium and were harvested at their respective mid-exponential growth phase. All other procedures regarding the DNA microarray experiments were performed essentially as described previously (Shafeeq et al., 2011b, 2012).

To confirm the polycistronic nature of the *ula2* operon, RT-PCR (reverse transcription PCR) reactions were performed as described before (Afzal et al., 2014) on the total RNA isolated from wild-type *S. pneumoniae* D39 grown in AM17 (10 mM ascorbic acid + M17) medium. For the fair comparison of PCR products, 100 ng of RNA and 20 ng of DNA were used.

### Microarray data analysis

DNA microarray data were analyzed as previously described (Shafeeq et al., 2011a,b). Briefly, microarray slide images were scanned using GenPix Pro 6.1 (MSD analytical technologies). Processing and normalization (LOWESS spotpin-based) of slides were done with the in-house developed *MicroPrep* software. DNA microarray data were obtained from independent biological replicates hybridized to glass slides, of which one was a dye swap. Expression ratios were calculated from the measurements of at least five spots. Differential expression tests were performed on expression ratios with a local copy of the Cyber-T implementation of a variant of the *t*-test. False discovery rates were calculated as described (Van Hijum et al., 2005). A gene was considered differentially expressed when *p*-value was <0.001 and false discovery rate was <0.05 and when at least five measurements were available. For the identification of differentially expressed genes, only genes with a fold change >2 were selected. Microarray data have been submitted to GEO (Gene Expression Omnibus) under the accession number GSE64107.

## Results

### Ascorbic acid-dependent gene regulation in *S. pneumoniae*

To elucidate the effect of ascorbic acid on the transcriptional responses of *S. pneumoniae* D39 strain, we compared the transcriptome of D39 wild-type grown in AM17 (10 mM ascorbic acid + M17) to that grown in M17 medium (no added sugar). The expression of several genes and operons with diverse functions were altered in the presence of ascorbic acid (Table 3). As expected, the expression of the *ula* operon was highly upregulated in the presence of ascorbic acid. In a previous study, we have shown that the expression of the *ula* operon is activated by the transcriptional regulator UlaR in the presence of ascorbic acid (Afzal et al., 2015). Therefore, the altered expression of the *ula* operon indicates that the conditions used for our transcriptome studies are suitable to explore the ascorbic acid-specific gene expression. The AdcR regulon was also upregulated in our microarray experiment. The AdcR regulon consists of zinc-dependent ABC transporters (AdcCBA), the zinc-dependent transcriptional repressor (AdcR), adhesion lipoprotein (LmB/AdcAII) and pneumococcal histidine triad (Pht) family proteins (PhtA, PhtB, PhtD, and PhtE). This regulon is shown to be involved in zinc transport and the transcriptional repressor AdcR represses its expression in the presence of zinc (Shafeeq et al., 2011a). The upregulation of the AdcR regulon in the presence of ascorbic acid suggests that the presence of ascorbic acid in the medium may cause zinc starvation.

**Table 3:** **Summary of transcriptome comparison of *S. pneumoniae* strain D39 wild-type grown in AM17 (10 mM ascorbic acid + M17) and in M17**.

**D39 tag^a^**	**Function^b^**	**Ratio^c^**
*spd_0167*	3,4-Dihydroxy-2-butanone 4-phosphate synthase/GTP cyclohydrolase II, RibB	2.0
*spd_0168*	Riboflavin synthase, alpha subunit, RibE	2.1
*spd_0169*	Riboflavin biosynthesis protein, RibD	2.1
*spd_0262*	PTS system, mannose/fructose/sorbose family protein, IID component	−2.2
*spd_0502*	PTS system, beta-glucosides-specific IIABC components	−2.3
*spd_0503*	6-Phospho-beta-glucosidase, BglA-2	−2.9
*spd_0773*	PTS system, fructose specific IIABC components	−2.4
*spd_0888*	Adhesion lipoprotein, AdcAII/LmB	3.2
*spd_0889*	Pneumococcal histidine triad protein D, PhtD	3.6
*spd_0890*	Pneumococcal histidine triad protein E, PhtE	4.4
*spd_1038*	Pneumococcal histidine triad protein A, PhtA	3.9
*spd_1053*	Galactose-6-phosphate isomerase, LacA subunit, LacA	2.5
*spd_1295*	Hemolysin	−4.6
*spd_1664*	PTS system, trehalose-specific IIABC components	−2.1
*spd_1839*	Transketolase, Tkt	4.7
*spd_1840*	L-ascorbate 6-phosphate lactonase, UlaG	35.5
*spd_1841*	Transcriptional regulator, UlaR	14.9
*spd_1842*	L-ribulose-5-phosphate 4-epimerase, UlaF/AraD	11.6
*spd_1843*	L-xylulose 5-phosphate 3-epimerase, UlaE	28.2
*spd_1844*	3-keto-L-gulonate-6-phosphate decarboxylase, UlaD	37.6
*spd_1845*	Ascorbate-specific PTS system, IIA component, UlaC	25.6
*spd_1846*	Ascorbate-specific PTS system, IIB component, UlaB	20.5
*spd_1847*	Ascorbate-specific PTS system, IIC component, UlaA	5.2
*spd_1957*	Transketolase, C-terminal subunit, TktC	2.3
*spd_1958*	Transketolase N-terminal subunit, TktN	3.0
*spd_1959*	PTS system, IIC component, putative, UlaA2	3.2
*spd_1960*	PTS system, IIB component, putative, UlaB2	2.3
*spd_1961*	Transcriptional regulator, UlaR2	2.4
*spd_1997*	Zinc ABC transporter, zinc-binding lipoprotein, AdcA	1.6
*spd_1999*	Zinc ABC transporter, ATP-binding protein, AdcC	1.6
*spd_2000*	*adc* operon repressor AdcR	2.1
*spd_2011*	Glycerol uptake facilitator protein, GlpF	−2.6
*spd_2012*	Alpha-glycerophosphate oxidase, GlpO	−4.2
*spd_2013*	Glycerol kinase, GlpK	−4.5

a *Gene numbers refer to D39 locus tags*.

b *D39 annotation/TIGR4 annotation (Lanie et al., 2007)*.

c *Ratio represents the fold increase/decrease in the expression of genes in D39 wild-type grown in AM17 compared to that in M17*.

The expression of the *ribDEBH* (*spd_0166-69*) operon was also upregulated in our microarray analysis. This operon is putatively involved in riboflavin metabolism and utilization of ribulose 5-phospate produced from L-ascorbate through the pentose phosphate pathway. Expression of the genes putatively involved in mannose, fructose, sorbose, β-glucosides, and trehalose uptake and utilization was also altered in our ascorbic acid-dependent microarray. An operon (*spd_2011-13*) consisting of glycerol uptake and utilization genes was downregulated in the presence of ascorbic acid. It has been shown that ascorbic acid causes inhibition of transport of these sugars in *E. coli* (Loewen and Richter, 1983). Hemolysin (*spd-1295*) was also downregulated about five times in the presence of ascorbic acid. A PTS operon (*spd_1957-61*) was upregulated in our microarray analysis. This operon has sequence homology with the *ula* operon of *S. pneumoniae*. Based on its sequence homology with the *ula* operon, we denoted this operon here as the *ula2* operon Upregulation of the *ula2* operon in the presence of ascorbic acid and its sequence homology with the *ula* operon may suggest its putative involvement in ascorbic acid utilization and metabolism. Therefore, we decided to further explore the organization and regulation of this operon in the presence of ascorbic acid.

### Organization of the *ula2* operon in *S. pneumoniae* D39

**Figure 1:**
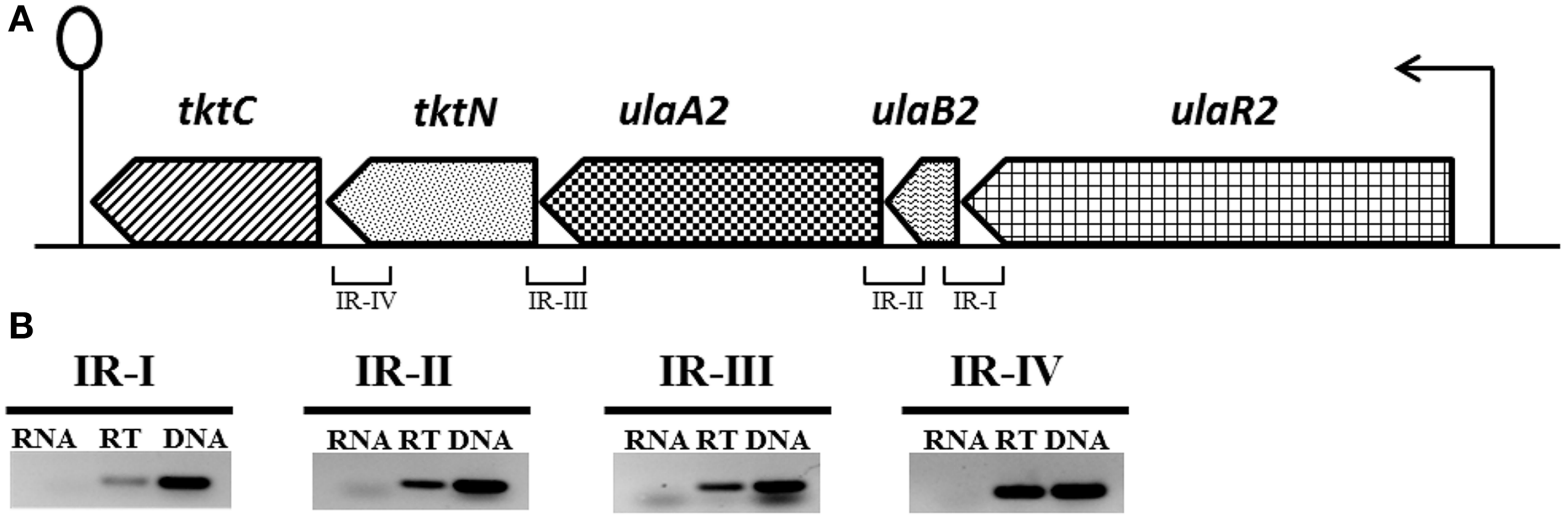
**(A)** Organization of the *ula2* operon in *S. pneumoniae* D39. Lollipop structure represents the putative transcriptional terminator while black arrows indicate the promoter regions. See text for further details. We take 1 kb = 2.5 cm here for the representation of gene size. **(B)** Reverse transcriptase (RT) PCR analysis to confirm the polycistronic nature of the *ula2* operon in *S. pneumoniae* D39. RT-PCR was performed on the total RNA isolated from D39 wild-type grown in AM17 (10 mM ascorbic acid + M17) medium with (RT) and without (RNA) reverse transcriptase treatment using the IR-I, IR-II, IR-III, and IR-IV intergenic region primer pairs. DNA was used as a positive control.

The *ula2* operon consists of five genes in *S. pneumoniae* D39 (Figure 1A), which encode an ascorbic acid-specific PTS (*ulaB2A2*: *spd_1959-60*), a transketolase N-terminal subunit (*tktN*: *spd_1958*) and a transketolase C-terminal subunit (*tktC: spd_1957*). *ulaB2* has 37% sequence identity to *ulaB* and *ulaA2* has 29% sequence identity to *ulaA* of the *ula* operon. Similarly, *tktC* and *tktN* have 24 and 35% identity to *tkt* of the *ula* operon.

RT-PCR using the primer pairs mentioned in Table 2 was performed to confirm that the *ula2* operon is transcribed as a single transcriptional unit. RT-PCR data showed that the *ula2* operon is transcribed as one transcriptional unit (Figure 1B).

### Ascorbic acid induces the *ula2* operon, while glucose, like other tested sugars, diminishes the expression of the *ula2* operon

**Figure 2:**
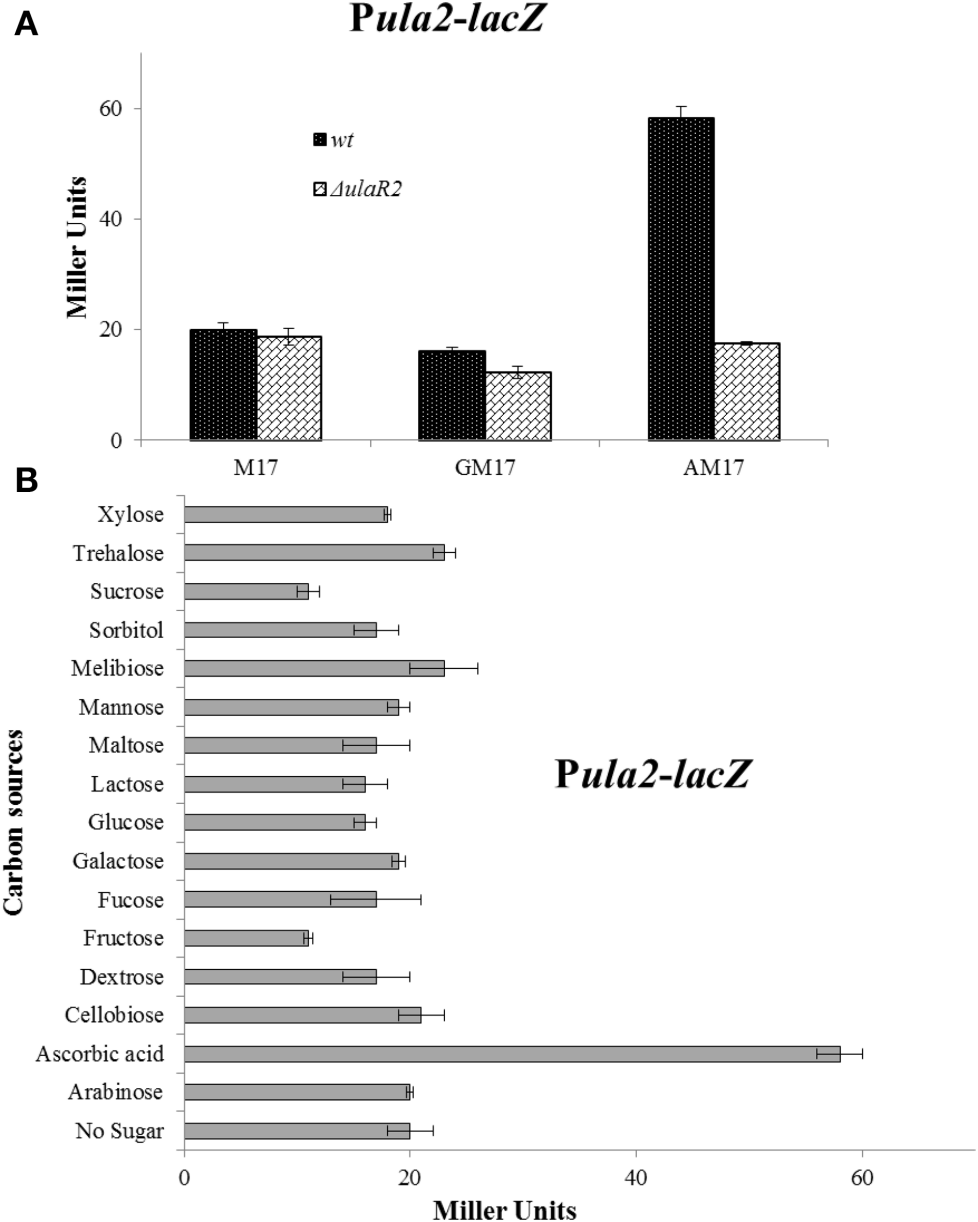
**(A)** Expression levels (in Miller units) of P*ula2-lacZ* in D39 wild-type and Δ*ulaR2* grown in M17 (without any sugar), GM17 (10 mM Glucose + M17) and AM17 (10 mM ascorbic acid + M17) medium. Standard deviation of three independent experiments is indicated in bars. **(B)** Expression levels (in Miller units) of P*ula2-lacZ* in D39 wild-type grown in M17 medium with different added sugars (10 mM). Standard deviation of three independent experiments is indicated in bars. The results of One-Way ANOVA are provided in the supplementary data (S1).

To verify that the altered expression of the *ula2* operon in our microarray analysis is due to the specific effect of ascorbic acid in the medium, we made a transcriptional *lacZ*-fusion to the promoter region of the *ula2* operon (P*ula2*) and transformed it into *S. pneumoniae* D39 wild type. The wild-type strain containing P*ula2-lacZ* was grown in M17 medium supplemented with 10 mM of different carbon sources (arabinose, ascorbic acid, cellobiose, dextrose, fructose, fucose, galactose, glucose, lactose, maltose, mannose, melibiose, sorbitol, sucrose, trehalose, and xylose) and β-galactosidase assays were performed. The expression of P*ula2-lacZ* was highest in the presence of ascorbic acid and lowest in the presence of all other sugars including glucose (Figure 2B). This data confirms our microarray results and also shows that ascorbic acid activates the expression of the *ula2* operon, while other tested carbon sources do not play a role in the activation of the *ula2* operon.

### UlaR2 acts as a transcriptional activator of the *ula2* operon in the presence of ascorbic acid in *S. pneumoniae*

The gene for the DNA-binding transcriptional regulator UlaR2 is present in the *ula2* operon. The presence of *ulaR2* in the *ula2* operon might be an indication of its involvement in the regulation of the *ula2* operon. To demonstrate the role of UlaR2 in the regulation of the *ula2* operon, we created a markerless knockout of *ulaR2* in *S. pneumoniae* D39 and introduced P*ula2-lacZ* in the Δ*ulaR2* strain to perform the β-galactosidase assay. Our β-galactosidase assay data show that expression of P*ula2-lacZ* was abolished in the Δ*ulaR2* strain compared to the wild-type strain even in the presence of ascorbic acid (Figure 2A). This data suggests the role of *ulaR2* as a transcriptional activator of the *ula2* operon in the presence of ascorbic acid.

### DNA microarray analysis of the Δ*ulaR2* strain

To study the impact of the *ulaR2* deletion on the transcriptome of *S. pneumoniae*, the transcriptome of wild-type D39 was compared to that of D39 Δ*ulaR2* grown in AM17 (10 mM ascorbic acid + M17) medium. AM17 medium was used to perform transcriptome analysis, as our β-galactosidase assay with P*ula2-lacZ* in the Δ*ulaR2* strain suggests that UlaR2 activates the expression of its targets in the presence of ascorbic acid. The transcriptomic changes incurred in *S. pneumoniae* D39 due to the deletion of *ulaR2* in the presence of ascorbic acid are summarized in Table 4. *ulaR2* was nearly six-fold downregulated in Δ*ulaR2*, confirming the deletion of *ulaR2* in the Δ*ulaR2* strain. The *ula2* operon was the only operon that was significantly downregulated in the Δ*ulaR2* strain after applying the criteria of >2.0-fold difference as the threshold change and a *P*-value < 0.001 was chosen. Downregulation of the *ula2* operon in the Δ*ulaR2* strain confirms the role of UlaR2 as a transcriptional activator of the *ula2* operon in the presence of ascorbic acid and also suggests that the *ula2* operon is the only target of UlaR2. Moreover, this data is also in accordance with our β-galactosidase data mentioned above. We further decided to explore the regulatory site of UlaR2 in P*ula2*.

**Table 4:** **Summary of transcriptome comparison of *S. pneumoniae* Δ*ulaR2* strain with D39 wild-type grown in AM17 (10 mM ascorbic acid + M17)**.

**D39 tag^a^**	**Function^b^**	**Ratio^c^**
*spd_1957*	Transketolase, C-terminal subunit, TktC	−2.8
*spd_1958*	Transketolase N-terminal subunit, TktN	−2.9
*spd_1959*	PTS system, IIC component, putative, UlaA2	−3.6
*spd_1960*	PTS system, IIB component, putative, UlaB2	−1.6
*spd_1961*	Transcriptional regulator, UlaR2	−5.6

a *Gene numbers refer to D39 locus tags*.

b *D39 annotation/TIGR4 annotation (Lanie et al., 2007)*.

c *Ratio represents the fold decrease in the expression of genes in ΔulaR2 as compared to the wild-type*.

### Prediction of an UlaR2 regulatory site in P*ula2*

**Figure 3:**
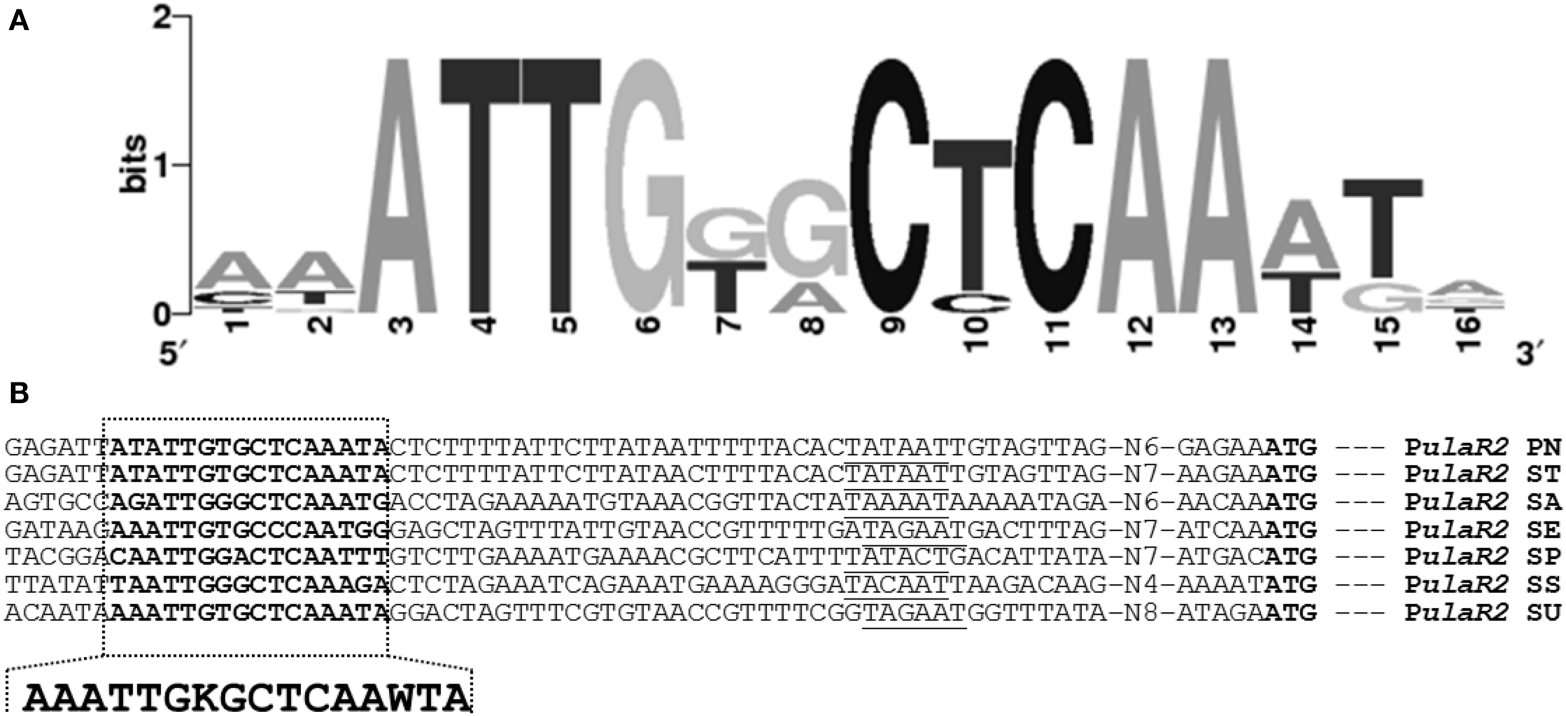
**Identification of the UlaR2 regulatory site in different streptococci**. **(A)** Weight matrix of the identified UlaR2 regulatory site in the P*ula2* of different streptococci. **(B)** Position of the UlaR2 regulatory site in the P*ula2* of different streptococci. PN, *S. pneumoniae;* ST, *S. mitis;* SA, *S. agalactiae;* SE, *S. equi*; SP, *S. pyogenes;* SS, *S. suis;* SU, *S. uberis*. Putative −10 sequences are underlined, translational start sites and putative UlaR2 regulatory sites are bold.

A 16-bp DNA sequence (5′-ATATTGTGCTCAAATA-3′) located upstream of the *ula2* operon of *S. pneumoniae* D39 was predicted by using a MEME motif sampler search (Bailey and Elkan, 1994) which might act as an UlaR2 regulatory site. This predicted UlaR2 regulatory site is found conserved in other sequenced strains of *S. pneumoniae* available in the KEGG database. *Streptococcus agalactiae*, *Streptococcus equi*, *Streptococcus mitis*, *Streptococcus pyogenes*, *Streptococcus suis*, and *Streptococcus uberis* also encode a putative *ula2* operon with a similar gene composition as in *S. pneumoniae* (Figure 3A). To find the conservation of the UlaR2 regulatory site in these streptococci, we analyzed the *ula2* promoter region in these species for the presence of an UlaR2 regulatory site. The analysis revealed that the UlaR2 regulatory site is highly conserved in these streptococci. We further generated a weight matrix of a 16-bp wide UlaR2 regulatory site (5′-AACACARRMCTGTGTK-3′) with the help of Genome 2D Software (Baerends et al., 2004) by aligning the predicted UlaR2 regulatory sequences in the P*ula2* of *S. agalactiae*, *S. equi*, *S. mitis*, *S. pyogenes*, *S. suis*, and *S. uberis* with that of *S. pneumoniae* (Figure 3B). To find more targets of UlaR2 in the D39 genome, we performed a genome-wide search with the pneumococcal UlaR2 regulatory site by using Genome 2D software (Baerends et al., 2004). We could not find any other stretch of DNA similar to the UlaR2 regulatory site in the D39 genome, suggesting that the *ula2* operon is the only target of UlaR2. This observation is also consistent with our transcriptome analysis with Δ*ulaR2*.

### Ascorbic acid activates/derepresses the expression of the AdcR regulon by decreasing the zinc concentration

**Figure 4:**
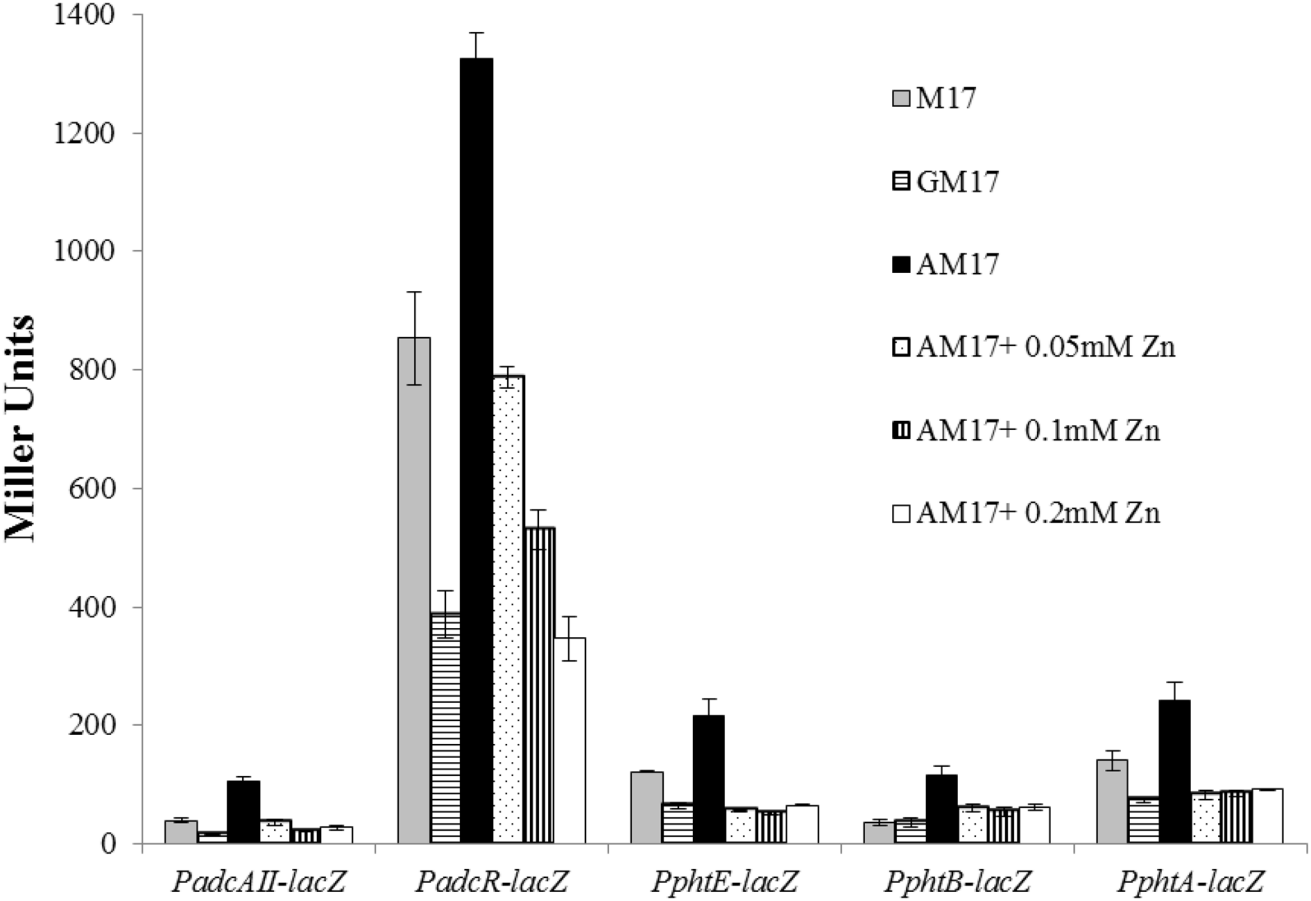
**Expression levels (in Miller units) of P*adcAII-lacZ*, P*phtB-lacZ*, P*phtE-lacZ*, P*phtA-lacZ*, and P*adcR-lacZ* in D39 wild-type grown in M17 (without any sugar), GM17 (10 mM Glucose + M17) and AM17 (10 mM ascorbic acid + M17) with the addition of increasing concentrations of zinc**. Standard deviation of three independent experiments is indicated in bars.

In our microarray analysis in the presence of ascorbic acid, expression of the AdcR regulon was upregulated. This might suggest the role of ascorbic acid in the regulation of the AdcR regulon. AdcR is a zinc-dependent transcriptional regulator that represses the expression of the AdcR regulon (which consists of genes that encode zinc transport and Pht-family proteins) in the presence of zinc (Shafeeq et al., 2011a, 2013). To study the role of ascorbic acid in the regulation of the AdcR regulon, we tested promoter-*lacZ* fusions of the genes that were upregulated in our microarray experiment in the presence of ascorbic acid (*adcAII-phtD*, *phtA*, *phtB*, *phtE*, and *adcRCBA*). The expression of these promoters was significantly higher in AM17 (10 mM ascorbic acid + M17) medium compared to M17 (no added sugar) and GM17 (10 mM glucose + M17) medium, confirming our microarray results (Figure 4). We further studied the role of zinc in the regulation of the AdcR regulon in the presence of ascorbic acid. We added various concentrations of zinc (0.05, 0.1, and 0.2 mM zinc) in AM17 medium (10 mM ascorbic acid + M17). Addition of zinc to the medium in the presence of ascorbic acid led to the repression of the following genes (*adcAII-phtD*, *phtA*, *phtB*, *phtE*, and *adcRCBA*) to the expression level without ascorbic acid (Figure 4). This data suggests that the presence of ascorbic acid in the medium causes zinc limitation in the cell.

To further investigate the role of ascorbic acid in zinc limitation, ICP-MS analysis was performed on the cells grown with and without 10 mM ascorbic acid in the medium (Table 5). Our ICP-MS analysis showed that the intracellular concentration of zinc decreases to less than half in the presence of ascorbic acid in the medium (Table 5). The concentration of zinc was 12 μg/g dry mass of cells grown in AM17, whereas this concentration was 28 μg/g dry mass of cells grown in M17 (without any added sugar). This data confirms that the altered expression of the AdcR regulon is due to zinc limitation in the medium caused by ascorbic acid. The levels of cobalt and nickel were below the detection level. Similarly, the manganese concentration was four times lower and the iron concentration was about three times lower in the presence of ascorbic acid. We could not observe any significant change in the expression of manganese- and iron-responsive genes in our microarray in the presence of ascorbic acid. This suggests that the concentrations of manganese and iron in the medium are already high enough to repress the expression of manganese-/iron-dependent genes. We also checked the concentration of metal ions in AM17, M17, and GM17 media by ICP-MS analysis. No difference in the concentration of metal ions, including zinc, was observed among these media (data not shown).

**Table 5:** **ICP-MS analysis of the cells grown in M17, GM17 (10 mM Glucose + M17) and AM17 (10 mM Ascorbic acid + M17) medium**.

**Concentrations (μg) of various metal ions/g of dry weight of cells**					
**Medium**	**Zn^2+^**	**Mn^2+^**	**Fe^3+^**	**Ni^2+^**	**Co^2+^**
M17	28	22.2	138	NA	NA
GM17	26.7	19.2	142	NA	NA
AM17	12.5	5.5	55	NA	NA

## Discussion

The concentration of ascorbic acid in human blood plasma is about 15 mg/L whereas this concentration may increase up to 50–70 times in lungs, brain, lymph nodes, liver, spleen, pituitary gland, and retina. During infection, this ascorbic acid can be a very useful carbon source for *S. pneumoniae* (Oreopoulos et al., 1993; Hediger, 2002). Ascorbic acid-dependent expression of the *ula* operon in *S. pneumoniae* was explored in our previous study that highlights the presence of an ascorbic acid-specific *ula* system positively regulated by a transcriptional regulator UlaR (Afzal et al., 2015). Deletion of the ascorbic acid-specific PTS (*ulaA*) encoded by the *ula* operon, did not lead to the abolition of growth of *S. pneumoniae* in the presence of ascorbic acid (Bidossi et al., 2012). This might suggest the presence of another ascorbic acid PTS in *S. pneumoniae*. The current study is aimed to explore the global gene expression of *S. pneumoniae* in the presence of ascorbic acid to identify the second putative ascorbic acid system. The *ula* operon expression was highly upregulated in our microarray analysis in the presence of ascorbic acid, consistent with the results presented in our previous study (Afzal et al., 2015). Furthermore, we found another PTS operon (the *ula2* operon) consisting of five genes (*spd_1957*-*61*) that is upregulated in the presence of ascorbic acid. We further explored the regulation of this operon in the presence of ascorbic acid and demonstrated that transcriptional regulator UlaR2 acts as a transcriptional activator of the *ula2* operon.

Our bioinformatic analysis shows that the *ula2* operon shares sequence homology with the ascorbate system in *K. pneumoniae* and *E. coli*, which has been shown to be involved in the transport and metabolism of ascorbic acid (Campos et al., 2008). However, there are some differences between them. The number of genes in the second ascorbic acid system varies among these bacteria. The *ula2* operon is composed of five genes in *S. pneumoniae* and regulated by a transcriptional activator, UlaR2, whereas *yiaK-S* consists of eight genes (*yiaKLX1X2PQRS*) that are organized in one operon in *K. pneumoniae* and regulated by a transcriptional repressor YiaJ. Similarly, *E. coli* also possesses the *yiaK-S* operon consisting of nine genes (*yiaKLMNOPQRS*) that is also regulated by a transcriptional repressor YiaJ and this second system has been shown to be involved in the ascorbic acid transport and metabolism (Campos et al., 2007). *yiaMNO* encodes the tripartite ATP-independent periplasmic transporter and has been reported to recognize L-xylulose as its substrate in *E. coli* (Plantinga et al., 2004). Another study using different techniques demonstrated that 2,3-diketogulonate, the breakdown product of L-ascorbate, is the substrate of *yiaMNO* in *E. coli* (Thomas et al., 2006). *yiaX2* encodes a secondary transport protein and *yiaX1* codes for a chemotaxis-like protein in *K. pneumoniae*. Similarly, *ula2* operon contains *ulaB2A2* as the transport genes in *S. pneumoniae*. The alternative presence of *ulaB2A2*, *yiaMNO*, and *yiaX2* with different substrate specificities and the presence of *yiaX1* suggest the extent of variation this system can exhibit and this diversification may suggest that this system is beneficial only under certain conditions.

In our transcriptome analysis, expression of several other sugar-specific genes was also altered in the presence of ascorbic acid. Ascorbic acid has been shown to inhibit the uptake of other sugars by various PTSs and this inhibition ranges from 0% (in case of mannitol PTS) to 90% (for glucose PTS) in *E. coli* (Loewen and Richter, 1983). This sugar uptake inhibition was not limited to the PTSs but also to the facilitated-diffusion uptake of glycerol. Other sugar transport systems were also inhibited to varying degrees (Loewen and Richter, 1983). The riboflavin metabolism operon *ribDEBH* (*spd_0166-69*) was also upregulated in ascorbic acid metabolism. RibH (*spd_0166*) is a 6,7-dimethyl-8-ribityllumazine synthase and RibB (*spd_0167*) is a 3,4-dihydroxy-2-butanone 4-phosphate synthase/GTP cyclohydrolase II that converts ribulose 5-phosphate into 3,4-dihydroxy-2-butanone 4-phosphate. RibE (*spd_0168*) is the alpha subunit of riboflavin synthase, whereas RibD (*spd_0169*) is the riboflavin biosynthesis protein carrying out the next steps of riboflavin metabolism in *S. pneumoniae*. Three transketolases (Tkt, TktN, and TktC), encoded by genes present in the *ula* and *ula2* operons, are also part of the pentose phosphate pathway and are involved in the subsequent reactions on the intermediates produced in this pathway. The upregulation of this operon in the presence of ascorbic acid suggests that ribulose 5-phosphate produced from ascorbic acid is utilized by *S. pneumoniae* for the biosynthesis and metabolism of riboflavin.

The presence of ascorbic acid in the medium also resulted in the upregulation of the AdcR regulon, consisting of zinc-responsive genes (*adcRCBA adcAII*, *phtA*, *phtB*, *phtD*, and *phtE*) that are involved in zinc transport and virulence (Reyes-Caballero et al., 2010). These genes are negatively regulated by the transcriptional repressor AdcR in the presence of zinc (Shafeeq et al., 2011a). Our ICP-MS analysis showed that the intracellular concentration of zinc decreased about twofold in the presence of ascorbic acid, suggesting that the altered expression of the AdcR regulon in the presence of ascorbic acid is due to zinc starvation in the cell caused by ascorbic acid. Ascorbic acid has already been shown to chelate iron and lead (Lynch and Cook, 1980; Simon and Hudes, 1999). The zinc starvation in the cell caused by ascorbic acid may be due to its metal chelating ability. The intracellular concentration of iron and manganese was also decreased three and four times, respectively, in the presence of ascorbic acid in the medium. However, we could not observe an effect on the expression of manganese- and iron-dependent genes in our ascorbic acid microarray, suggesting that cells still have a high enough concentration of these metal ions for the unchanged expression of the manganese- and iron-dependent genes. A more sophisticated study (involving chemically-defined medium) might provide more insight into the mechanisms by which ascorbic acid causes the starvation of the metal ions inside the cell.

Our bioinformatic analysis showed that the UlaR2 in *S. pneumoniae* has PTS regulation domain (PRD). The PRD is the phosphorylatable regulatory domain present in bacterial transcriptional activators that usually activates the expression of genes and operons involved in the carbohydrate metabolism (van Tilbeurgh and Declerck, 2001). These transcriptional regulators contain two PRDs with highly conserved histidine residues (van Tilbeurgh and Declerck, 2001). The histidine residues in these PRDs are the targets of the PTS-catalyzed phosphorylation events (Rothe et al., 2012). In addition to PRD, UlaR2 also contains an HTH (Helix Turn Helix) domain, a PTS_EIIA-like and a PTS_EIIB-like domain in *S. pneumoniae*. Domain organization of UlaR2 is similar to transcriptional regulator UlaR which has two PRDs, an HTH domain and a PTS_EIIB domain (Afzal et al., 2015) and CelR in *S. pneumoniae* which also has an HTH, two PRDs in addition to an EIIA and an EIIB domain (Shafeeq et al., 2011c). The presence of PRD in UlaR2 suggests that UlaR2 in *S. pneumoniae* is a PRD-regulated transcriptional regulator belonging to the PRD-containing transcriptional regulators family.

CcpA (Carbon catabolite protein A) is a master transcriptional regulator that represses the expression of genes other than those involved in the transport and metabolism of a preferred carbon source i.e., glucose, through a mechanism called CCR (Carbon Catabolite Repression) (Lulko et al., 2007; Zomer et al., 2007). In this study, we have studied the role of different sugars on the expression of P*ula2-lacZ* and observed that the expression of P*ula2-lacZ* was highest in the presence of ascorbic acid compared to other tested sugars. To determine the role of CcpA in the repression of the *ula2* operon caused by glucose, we analyzed the promoter region of *ulaR2* for a putative *cre* (catabolite repression element) box. We could not find any *cre* box in the promoter region of the *ula2* operon. The absence of a *cre* box in P*ula2* suggests that CcpA may not be involved in the regulation of the *ula2* operon. These findings are consistent with the observations mentioned in a previous study (Carvalho et al., 2011) where no effect on the expression of *ula2* operon was observed in a Δ*ccpA* strain in the presence of galactose and glucose.

The two ascorbic acid systems (*ula* and *ula2*) are highly conserved in other sequenced strains of *S. pneumoniae* available in the KEGG database. These *ula* and *ula2* operons are present in a similar organization as in the D39 strain in these pneumococcal strains, except for the G54 strain that has an additional PTS subunit IIA present in the *ula2* operon. The conservation and similar organization of the *ula* and *ula2* operons in these pneumococcal strains indicate a similar pattern of regulation of the *ula* operon by UlaR and the *ula2* operon by UlaR2 in the presence of ascorbic acid. Moreover, conservation of these two systems in other pneumococcal strains suggests the important physiological function of these two systems in the life style of *S. pneumoniae*. Further studies involving animal models will highlight the importance of these two systems in the pathogenicity of *S. pneumoniae*.

### Conflict of interest statement

The authors declare that the research was conducted in the absence of any commercial or financial relationships that could be construed as a potential conflict of interest.
